# Do microplastics impair male dominance interactions in fish? A test of the vector hypothesis

**DOI:** 10.1002/ece3.8620

**Published:** 2022-02-16

**Authors:** Ally Swank, Kadijah Blevins, Abby Bourne, Jessica Ward

**Affiliations:** ^1^ 5666 Department of Biology Ball State University Muncie Indiana USA; ^2^ Present address: 1383 Department of Biological Sciences Auburn University Auburn Alabama USA

**Keywords:** aggression, endocrine‐disrupting chemical, estrogen, male–male competition, multiple stressors, sexual selection

## Abstract

Microplastics (MPs) are widespread in aquatic environments and have become a critical environmental issue in recent years due to their adverse impacts on the physiology, reproduction, and survival of aquatic animals. Exposure to MPs also has the potential to induce sub‐lethal behavioral changes that can affect individual fitness, but these effects are understudied. Many plastic additives introduced during the manufacture of MPs are known endocrine‐disrupting chemicals (EDCs) that mimic the action of natural hormones, alter sexual and competitive behavior, and impair reproductive success in fish. In addition, EDCs and other aquatic contaminants may adhere to MPs in the environment, the latter of which may serve as transport vectors for these compounds (i.e., *the vector hypothesis*). In this study, we staged territorial contests between control males, and males exposed to virgin MP particles or to MPs previously immersed in one of two environmentally relevant concentrations of 17‐alpha ethinyl estradiol (EE2; 5 ng/L and 25 ng/L) to evaluate the independent and synergistic effects of exposure to MPs and a common environmental estrogen on male–male aggression and competitive territory acquisition in a freshwater fish, *Pimephales promelas*. Short‐term (30 days) dietary exposure to MPs did not impair the ability of males to successfully compete for and obtain a breeding territory. Overall levels of aggression in control and exposed males were also similar across trial series. These results help to fill a critical knowledge gap regarding the direct and indirect (vector‐borne) effects of MPs on the reproductive behavior of aquatic vertebrates in freshwater systems.

## Graphical abstract

This paper evaluated the effects of microplastic exposure on male–male territorial aggression and dominance in fish. Short‐term exposure to microplastics, alone or in association with another common aquatic contaminant, 17‐alpha ethinyl estradiol, did not significantly impair the ability of male fathead minnows to compete for a territory.

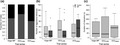

## 1 INTRODUCTION

Microplastics (MPs), informally defined as particles with diameter of <5 mm, are sourced from the breakdown of larger plastic items such as containers, synthetic clothing, and other commercial and industrial products (Foley et al., 2018). These contaminants are ubiquitous in freshwater and marine environments (Barnes et al., 2009; McNeish et al., 2018) and have become a critical environmental issue in recent years due to documented adverse impacts on the physiology, reproduction, and survival of animals (Andrady, 2011; Eerkes‐Medrano et al., 2015). In the environment, MPs enter into food webs via ingestion, respiratory intake, and adherence (Ašmonaitė & Almroth, 2019; Scherer et al., 2017). Smaller particles may be more easily absorbed and/or translocated within organisms and are associated with higher potential for trophic transfer and bioaccumulation (Batel et al., 2016; Brennecke et al., 2015).

Knowledge regarding the biological impacts of MPs has increased rapidly in recent years, and several knowledge gaps have been identified with explicit requests for research prioritization (Foley et al., 2018; Wagner et al., 2014). Among the most pressing of these are studies that examine the interactive effects of microplastics and other contaminant stressors on the behavior of organisms. In aquatic environments, exposure to endocrine disrupting chemicals (EDCs) that mimic natural hormones has well‐documented effects on the physiology and behavior of fishes (Arukwe, 2001; Kolpin et al., 2002; Mills & Chichester, 2005; Soffker & Tyler, 2012). Specifically, EDCs are well known to impair the expression of sexually selected traits in males (Borg, 1994; Mayer et al., 2004) and alter the outcomes of conspecific social interactions (e.g., male–male dominance interactions and male–female sexual interactions; Martinovic et al., 2007; Saaristo et al., 2009; Saaristo et al., 2010). As a result, EDC exposure can not only influence individual reproductive success but also population viability (Jobling & Tyler, 2003; Kidd et al., 2007).

Due to their chemical composition, MPs may accumulate aquatic contaminants, including EDCs, and serve as vectors of transport for these compounds (Endo & Koelmans, 2016), with adverse effects on the reproductive behavior and success of fish and other aquatic organisms. Plastics additives also have known endocrine‐disrupting properties that can be discharged into the bodies of aquatic organisms, amplifying this toxic effect (Liu et al., 2019). To date, few studies have explicitly tested the ‘vector hypothesis’ (Wagner et al., 2014) to determine the extent to which the behavior of fish and other aquatic organisms may be affected by EDCs and other contaminants that adhere to MP particles in the environment and desorb within the intestine of affected individuals. As a step toward filling this critical knowledge gap, in this study we evaluated the biological effects of virgin MP particles, and those exposed to a common environmental estrogen, 17‐alpha ethinyl estradiol (EE2), on dominance and male–male aggression in the fathead minnow, *Pimephales promelas*. *Pimephales promelas* is a small‐bodied North American freshwater fish with a widespread distribution (Page & Burr, 2011). This generalist species inhabits streams, rivers, and lakes subject to influxes of microplastics and other contaminants, and is commonly used as a fish model to study of the effects of contaminants on behavior and other biological endpoints because it is easy to maintain in the lab, tolerant of a wide range of water quality characteristics, and has well‐defined reproductive and development cycles (Ankley & Villeneuve, 2006; Burns et al., 2016). Despite growing evidence of the adverse biological effects of MPs on aquatic animals, to our knowledge this is the first study to evaluate the effects of MPs on reproductive behavior critical to reproductive success in fish.

## 2 METHODS

### 2.1 Study species, housing, and maintenance

In nature, male *P*. *promelas* aggressively compete for territories underneath logs or floating materials that serve as nest sites. Females deposit eggs in a single layer to the underside of the substrate, and the male defends the nest from potential predators, including conspecifics (Clement et al., 2004; TerMarsch & Ward, 2020; Unger, 1983). Because the ability of a male to obtain a territory is a prerequisite to mating, contaminant‐induced changes in male aggression and competitive ability have the potential to reduce reproductive success.

Behavioral assays took place between October 2020 and May 2021. Mature, male *P*. *promelas* (>6 months old) were obtained from a laboratory culturing facility (Aquatic Biosystems, Fort Collins, CO) at regular intervals and maintained for use in experiments in a 400‐gallon living stream unit (model LS‐120, Frigid Units). The housing unit was fitted with two filters and additional air stones to ensure adequate oxygenation and water flow. The tank contained four mesh baskets that served as habitat structure within the holding tank; each basket contained ~50 juvenile *P*. *promelas* that were used for other experiments. A maximum of 50 mature males were housed in the living stream at one time, and all were kept throughout the experiment under summer breeding conditions (21–23°C and a 16:8 h light:dark photoperiod). Fish were fed newly hatched brine shrimp (*Artemia* spp.) twice daily *ad libitum*. To ensure the health and reproductive motivation of the fish, water quality measurements (alkalinity, hardness, nitrates, nitrites, and chlorine) were made on a weekly basis and temperature and total dissolved solids were assessed daily.

### 2.2 Microplastic particles and chemical solutions

Round, virgin polyethylene microspheres (300‐ to 350‐μm diameter; Cospheric, Santa Barbara, California) were used in this study. We used polyethylene because it is the most abundant microplastic found in aquatic habitats (Rochman et al., 2014) and previous studies have shown that the ingestion of polyethylene induces changes in the function of the endocrine system in male fish (Rochman et al., 2014). Before the experiment, MPs were prepared for use by enclosing the particles in 75‐micron nylon micromesh bags and immersing them in one of two environmentally relevant concentrations of EE2 (5 ng/L or 25 ng/L; Sigma Aldrich), or a clean water control. These concentrations of EE2 represent low and high environmental values reported in surface water samples of both freshwater and marine systems globally (Aris et al., 2014). The particles were soaked for 72 h to allow the EE2 to fully sorb to the MPs. This duration of time was deemed appropriate based on previous research showing that sorption rates of EE2 to microplastics plateaued after 48 h and then slowly increased by <1% until 96 h (Lu et al., 2020; Wu et al., 2015). Fresh EE2 solutions were made each day by adding 5 µL (EE2_LOW_) or 25 µL (EE2_HIGH_) of a common stock solution (1 µg/mL) to an appropriate volume of aged, aerated water, and the water was exchanged daily. After the 72‐h soaking period, the MPs were removed from solution, air‐dried, and aliquoted into single‐feeding vials by weight (12.5 mg of MPs, or ~500 MP particles). The vials were frozen at −20ºC and thawed just prior to use.

### 2.3 Preliminary study

To confirm that the MPs were naturally ingested while the fish foraged for brine shrimp, before starting the behavioral experiment we conducted a pilot study using a feeding protocol modified from previously published studies (Critchell & Hoogenboom, 2018; Mak et al., 2019; Rochman et al., 2014). Pilot exposures occurred for either 4 or 7 days (*n* = 10 fish for the 4‐day exposure and *n* = 8 for the 7‐day exposure), during which time the fish were fed freshly hatched brine shrimp and aliquoted MPs *ad libitum* twice daily (morning and late afternoon). To aid in identification of MPs during dissection, blue virgin polyethylene particles were used for this preliminary experiment. Male test subjects were housed in 38‐L charcoal‐filtered home tanks (50.8 × 25.4 × 30.5 cm) in groups of four. At the start of a dietary exposure event, the fish were removed from their home tank and introduced to a 6‐L exposure tank containing 5 L of aged, aerated fresh water. Approximately 1 mL of live, freshly hatched brine shrimp was mixed with the aliquoted MPs and added to the tank. Thus, feeding males were exposed to MPs at a concentration of ~100 MPs/L. We selected this level of exposure based on the highest reported estimates of MP concentrations in surface water samples of natural waterways (range, <1–100 MPs/L; Burns & Boxall, 2018; Cunningham & Sigwart, 2019; Leslie et al., 2017). The fish were permitted to forage freely for 30 min, and the presence of an airstone ensured that the MPs remained suspended in the water column. The focal fish were removed from the exposure tank immediately following treatment and returned to their home tank. The water from the exposure tank was filtered through a micromesh sieve to capture any remaining MPs, and the tank was thoroughly rinsed.

At the end of the 4‐ or 7‐day exposure period, we euthanized the fish using an overdose of MS‐222 and dissected them in the morning following the last feeding (i.e., 16–20 h after the last feeding event). We recorded and compared the number of MPs found in the digestive tracts of fish after 4 or 7 days of exposure. Microplastic particles have a retention time of 72–96 h in *P*. *promelas* (Elizalde‐Velazquez et al., 2020); therefore, this comparison allowed us to determine whether the MPs were being egested or accumulating within the digestive tract. Microplastics have also been shown to translocate to other organs within the body following ingestion (Lu et al., 2016), so we also recorded the numbers of MP particles found in the body cavity or gills. In addition, the standard length (mm) and body weight (g) of each fish was recorded. The weight of the digestive tract was recorded for six fish in each group. Non‐parametric Mann–Whitney tests were used to analyze the data.

### 2.4 Behavioral experiment

Males used in the behavioral experiment were exposed to MPs or a control water equivalent for 30 days before undertaking behavioral tests. At the beginning of each exposure period, eight visually size‐matched males were removed from the stock tank. Each set of eight fish was divided into two groups: one of these groups was assigned to an exposure treatment (virgin, EE2_LOW_, or EE2_HIGH_) and the other served as a control group for that exposure group. Each group of four fish was housed in a 38‐L charcoal‐filtered aquarium (50.8 × 25.4 × 30.5 cm) containing four PVC shelters for enrichment. The four fish in a given tank were housed together for the entirety of the exposure period, but did not have visual access to other males in order to eliminate familiarity between competitors. Familiarity has been shown to reduce aggression and impact dominance hierarchies within fish populations (Ward & Hart, 2003). All males were permitted visual access to females in neighboring tanks to maintain reproductive motivation during the exposure period.

Test subjects were fed according to the same procedure described above for the preliminary study, with the exception that white MP particles were used. Although the color of MPs has been suggested to impact consumption rates in fish, McNeish et al. (2018) reported that *P*. *promelas* consume blue and white MPs at a similar rate. Control males underwent the same feeding protocol, but no MPs were added to their food. Visual barriers between the feeding tanks ensured that males in different treatments could not see each other while foraging. After 30 days of exposure, each individual from an MP‐exposure tank was paired with an individual from the corresponding control tank, resulting in a maximum of four trials per exposure replicate. Six replicate exposures were conducted for the virgin MP and EE2_LOW_ groups and five replicate exposures were conducted for the EE2_HIGH_ group. A total of 67 trials were conducted (control vs. virgin: *n* = 24, control vs. EE2_LOW_: *n* = 23, and control vs. EE2_HIGH_: *n* = 20), and each fish was only used in one trial. Of this total number of trials, nine virgin MP trials, five EE2_LOW_ trials, and one EE2_HIGH_ trial were discarded prior to behavioral scoring because they failed to meet inclusion criteria. Specifically, trials were discarded if a contest winner could not be declared or paired males differed in SL by >5 mm. In the case of one EE2_LOW_ trial, the camera position was such that detailed behavioral interactions could not be reliably scored. Therefore, this trial was retained for analyses of contest outcome (territory acquisition) only. Final sample sizes used for statistical analyses were as follows: control vs. virgin: *n* = 15, control vs. EE2_LOW_: *n* = 18, and control vs. EE2_HIGH_: *n* = 19.

### 2.5 Male–male territorial contests

**FIGURE 1:**
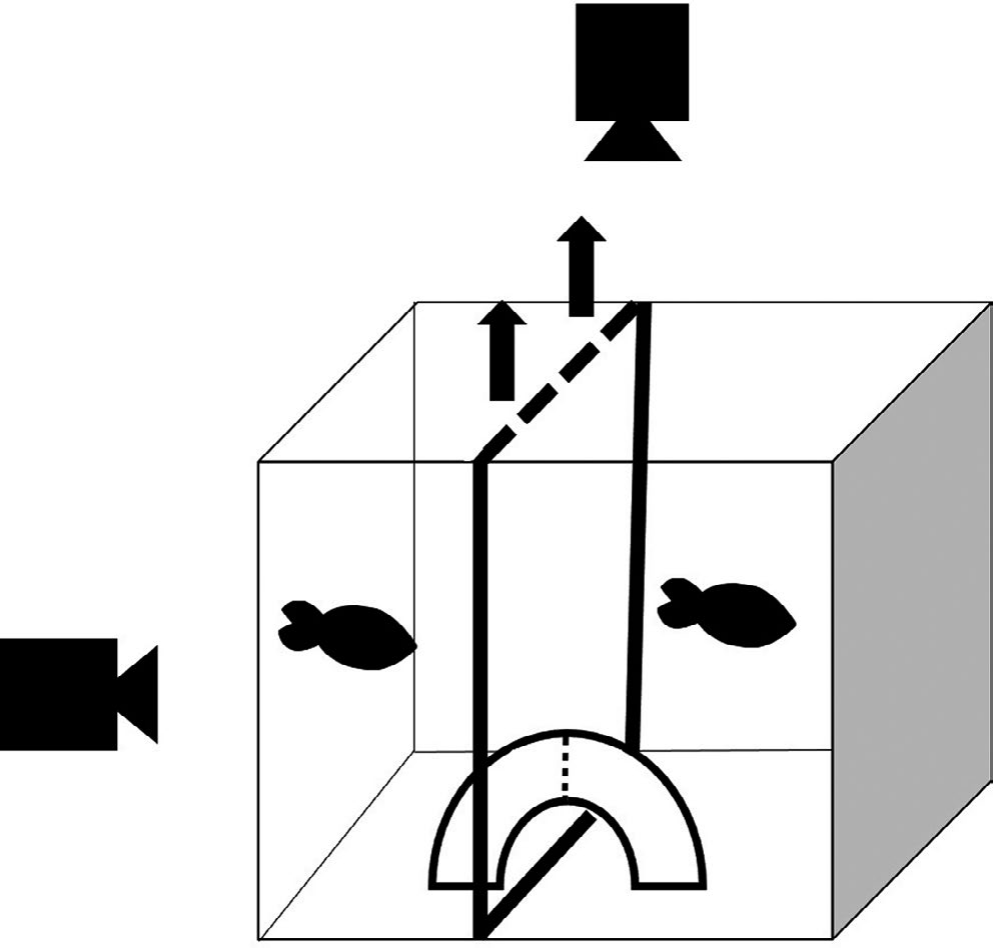
Experimental set up. Males were given 24 h to establish a territory on either side of a divider before testing. As indicated by the arrows, the divider was removed at the start of the interaction period, requiring males to compete for a single spawning territory

On day 31 of the experiment, we staged paired territorial contests between control and MP‐exposed males using procedures adapted from TerMarsch and Ward (2020). Briefly, trials were conducted in 38‐L tanks (50.8 × 25.4 × 30.5 cm) covered on the sides and back with pictures of underwater foliage to mimic the natural environment of the fish. The tanks were illuminated with a LED light source (Kessil, Richmond, CA). Each tank was divided in half via a permanent, opaque acrylic divider to create two testing arenas. Each arena was sub‐divided into two compartments of equal size (12.7 cm) using a removable acrylic divider. A quarter section of PVC pipe (7.6 cm diameter) was positioned against the central divider in each compartment and served as a spawning territory for each male. All trials were filmed using two GoPro Hero 5 cameras (San Matea, CA, USA) positioned 10 cm above and in front of the trial tanks (Figure 1).

Before a trial started, one visually size‐matched (SL difference <5 mm; Hudman & Gotelli, 2007) control male and exposed male (virgin, EE2_LOW_, or EE2_HIGH_) were selected from their home tanks, fin‐clipped for identification, and introduced to the test arena on either side of the arena divider. The positions of control and exposed males (left‐right) were randomized to prevent confounding effects of side bias. Males were given 24 h to acclimate to the trial tanks and to establish a territory, as evidenced by prolonged presence under the PVC shelter. A pretrial video recording of male behavior was made for 5 min to confirm that male displays defined as aggression only occurred only in response to a competitor, and were not spontaneously performed in non‐territorial contexts. At the end of the 5‐min pretrial period, the barrier was removed from the testing arena, and the two males were allowed to compete for a single nest site in the center of the arena created by the two quarter sections of PVC pipe. Interactions between the two males were recorded for 10 min (a 5‐min acclimation period after the removal of the divider and a subsequent 5‐min observation period of male–male aggression). Following the 15‐min (total) behavioral trial, the fish were monitored for 24 h, with snapshot observations made 6, 12, and 24 h after the trial started. During each snapshot observation, the identity of the male located underneath or immediately adjacent to the PVC shelter (i.e., exposed or control) was recorded. Shelter possession was recorded as “none” if neither or both males were under the shelter. A contest “winner” was declared if one of the two fish was found beneath the shelter during at least two of three snapshot observation periods. Immediately after the trial, both males were removed from the arena, euthanized via an overdose of MS‐222, and weighed and measured for standard length.

### 2.6 Behavioral variables scored and statistical analysis

We recorded the frequencies of aggressive behaviors performed by each male in each trial during the 5‐min baseline period before removal of the divider, and during the 5‐min trial interaction observation period after removal of the divider and subsequent 5‐min acclimation period. Aggressive behaviors were defined as follows: *Tail*‐*flicking* (quick, short movement of one individual's tail/caudal region); *head*‐*butting* (pushing of one's snout to any other part of the opponent's body); *charging* (quick movement of one fish toward the other); *biting* (open mouth‐to‐body contact between two opponents); *lateral display* (an individual turns its body lateral towards its opponent in a parallel orientation); *lateral display with contact* (an individual pushes its lateral trunk against its opponent in either a parallel or perpendicular orientation); and *blocking* (an individual blocks the opening to the shelter by turning its body lateral towards its opponent) (Phillips et al., 2009; Pyron & Beitinger, 1989; TerMarsch & Ward, 2020). We calculated the total number of aggressive behaviors performed by each male during each trial period; as well as the total duration of time spent engaged in aggressive interaction during the trial.

Initial screening indicated that the data violated parametric assumptions; therefore, we used generalized linear models with a negative binomial distribution and a log‐link function to compare the frequencies of individual behaviors performed by control and exposed males within trial series, as well as the total number of aggressive displays performed by interacting males (O’Hara & Kotze, 2010). This model corrects for the presence of zeros in a count dataset, which is common in behavioral studies. P values were Bonferroni‐corrected for multiple comparisons prior to interpretation. We compared the proportions of exposed and control males that won the territorial contest in each trial series using a two‐tailed binomial test of the null hypothesis that equal proportions (0.5) of males would win the contest. We used a generalized linear model to compare the overall level of aggressive intensity in trials across trial scenarios in terms of the total number of displays performed by both males during the trial. We used a non‐parametric Kruskal–Wallis test to compare the total duration of trial time that was spent in aggressive interactions across the three sets of trials. All analyses were conducted using SPSS ver 27 (IBM).

## 3 RESULTS

### 3.1 Preliminary dissections

One hundred percent of the fish in the preliminary experiment were found to contain MPs in their digestive tract (*n* = 8 and 10 for fish fed for 4 and 7 days, respectively). In addition, one fish fed for 4 days had two MP particles in the gills, and another contained two MP particles in the body cavity. The mean total number of MPs recovered from the 7‐day exposure group was 15% more than that recovered from the 4‐day exposure group (4 days: mean ±SD: 17.1 ± 12.3, vs. 7 days: 20.1 ± 14) but this difference was not statistically significant (*U* = 42, *p* = .90). Similarly, we did not find statistically significant differences between the 4‐day and 7‐day exposure groups in standard length (4 days: 48.86 ± 5.29 mm, vs. 7 days: 50.52 ± 2.46 mm; *U* = 40, *p* = 1.0) or weight (4 days: 2.44 ± 0.66 g, vs. 7 days: 2.66 ± 0.43 g; *U* = 44.5, *p* = .70). However, the weight of the digestive tract was significantly heavier in males exposed to microplastics for 7 days, compared to those exposed for 4 days (4 days: 0.12 ± 0.06 g, vs. 7 days: 0.21 ± 0.08 g; *U* = 32, *p* = .03).

### 3.2 Male–male contests

In the behavioral experiment, there was no difference between control and exposed males in terms of either standard length (mean ± SD: control: 58.59 ± 4.71 mm vs. exposed: 58.63 ± 4.28 mm; *n* = 52 males in each group) or body weight (control: 3.32 ± 0.83 g vs. exposed: 3.20 ± 0.73 g). No male performed any aggressive displays during the 5‐min pretrial observation period. However, aggressive interactions ensued between control and exposed males in most trials after the divider was pulled (virgin MPs: 13/15 trials; EE2_LOW_: 12/17 trials; EE2_HIGH_: 16/19 trials), indicating that our experiment was sufficient to elicit territorial behavior. The mean (±SD) pre‐trial and interaction frequencies of total behaviors performed for each of the trial series were as follows: virgin: 0 ± 0 vs. 22.03 ± 26.03; EE2_LOW_: 0 ± 0 vs. 29.62 ± 35.05; EE2_HIGH_: 0 ± 0 vs. 32.66 ± 33.87.

**FIGURE 2:**
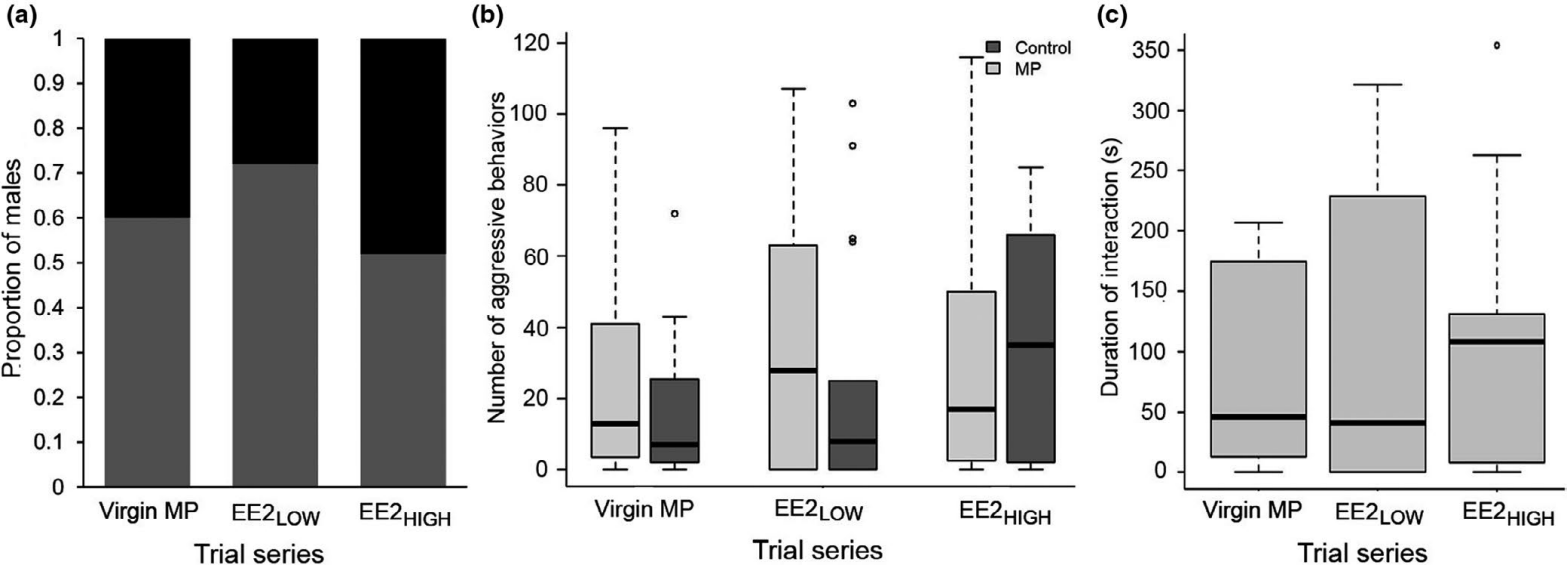
Aggressive behavior during male–male contests. (a) Relative proportions of control (gray) and MP‐exposed (black) males that won the territory in each trial series. (b) Boxplots showing total frequencies of aggressive displays performed by control (dark gray) and exposed (light gray) males (virgin MP, EE2_LOW_, EE2_HIGH_) in each trial series. (c) Boxplots showing total duration of aggressive interaction between males in each trial series. Control vs. virgin MP‐exposed males, *n* = 15 trials; vs. EE2_LOW_, *n* = 18 trials; vs. EE2_HIGH_, *n* = 19 trials. Behavioral interactions could not be reliably scored for one EE2_LOW_ pair; therefore, the sample size in panels (b) and (c) is 17

We did not find that MP‐exposed males won significantly fewer territorial contests than expected by chance in any trial series (binomial tests: *ps* > .05; Figure 2a). In the contest that paired a control male against a male exposed to virgin MPs, 9/15 trials (60%) were won by control males. Comparable results were observed in the trials that paired a control male against an exposed male in the EE2_LOW_ (13/18 males; 72%) and EE2_HIGH_ treatments (10/19 males; 53%). Within each trial series, the overall intensity of aggression between contestants was positively, significantly correlated (Spearman correlations: control vs. virgin: *r* = .882, *p* < .001; control vs. EE2_LOW_: *r* = .882, *p* < .001; control vs. EE2_HIGH_: .812, *p* < .001). Correspondingly, exposed and control males in each trial series competed for the shelter with a similar level of aggressive intensity (total number of aggressive displays: control vs. virgin MP: *χ* ^2^ = 1.38, df = 1, *p* = .24; control vs. EE2_LOW_: *χ* ^2^ = .87, *df* =1, *p*= .35; control vs. EE2_HIGH_: *χ* ^2^ = .22, *df* = 1, *p* = .64; Figure 2b). Subsequent inspection of the frequencies of individual aggressive displays also indicated that the relative use of threat (e.g., lateral display, tail flick, block) and fight (e.g., charge, headbutt, bite) displays performed by males was unaffected by dietary exposure to MPs (Table 1).

**TABLE 1:** Descriptive statistics and results of generalized linear models comparing the frequencies of aggressive displays performed by control males, and males exposed to virgin MPs or MPs immersed in EE2, during territorial contests

	Virgin MPs (*n *= 15)				EE2_LOW_ (*n* = 17)				EE2_HIGH_ (*n* = 19)			
	Mean (SD)		*χ* ^2^	*p*	Mean (SD)		*χ* ^2^	*p*	Mean (SD)		*χ* ^2^	*p*
	Control	Exposed			Control	Exposed			Control	Exposed		
Tailflick	5.13 (5.81)	11.80 (19.43)	4.56	.03	6.18 (11.22)	12.06 (19.32)	3.39	.07	10.89 (12.52)	9.67 (9.83)	0.12	.73
Headbutt	3.33 (6.85)	4.40 (4.90)	0.45	.50	8.41 (14.12)	10.53 (17.43)	0.39	.53	14.37 (17.93)	12.84 (17.25)	0.11	.74
Charge	NA	NA	—	—	0.24 (0.75)	0.59 (1.94)	1.80	.18	0.21 (0.54)	NA	—	—
Block	0.67 (1.59)	0.60 (1.84)	0.03	.86	0.29 (0.99)	1.88 (6.76)	9.88	**.002**	0.84 (2.52)	0.47 (1.17)	0.19	.28
Bite	2.20 (7.73)	0.93 (2.60)	3.13	.08	3.12 (6.25)	2.29 (4.52)	0.58	.45	1.95 (3.24)	2.16 (4.21)	0.07	.80
Lateral (contact)	0.73 (1.49)	1.20 (2.51)	0.87	.35	1.94 (3.73)	2.65 (4.17)	0.57	.45	3.11 (4.11)	2.74 (3.57)	0.11	.74
Lateral (no contact)	5.20 (4.59)	7.87 (10.01)	1.11	.29	4.47 (5.93)	4.65 (5.20)	0.01	.92	2.74 (3.60)	2.79 (3.97)	0.003	.96

Note

Significant *p* values after Bonferroni correction given in bold.

Abbreviations: EE2, 17‐alpha ethinyl estradiol; MP, microplastic particles; NA, not applicable—No behavior observed; SD, standard deviation.

Last, neither the total number of observed aggressive displays (*χ* ^2^ = 1.34, *df* = 2, *p *= .51) nor amount of time that males spent engaged in aggressive interactions (*H* = .029, *df* = 2, *p* = .98; Figure 2c) differed among the three trial series.

## 4 DISCUSSION

In this project, we evaluated the biological effects of MP particles, and those exposed to estrogen (i.e., the vector hypothesis) on dominance interactions and territorial success in a freshwater fish. Despite a wealth of studies documenting the adverse effects of MP exposure on the behavior of fish (Bour et al., 2020; Critchell & Hoogenboom, 2018; Mak et al., 2019; Pannetier et al., 2020; Rios‐Fuster et al., 2021; Yin et al., 2018), to our knowledge no prior studies have investigated the impacts of MPs on the outcomes of complex behaviors crucial to reproductive success. The males in our study (i) consumed MPs as a byproduct of normal feeding events, and (ii) readily engaged in aggressive contests over a limited resource (i.e., a breeding territory). However, in this study, exposure to MPs (alone or in association with estrogen) did not reduce levels of aggression in male contests or impair the ability of males to compete with non‐exposed males for a nest site.

The observed lack of an impact of MP exposure on the fish contrasts with previous studies that have demonstrated impairments in a variety of behaviors following MP ingestion, including swimming performance, activity, foraging, and locomotion (Carlos de Sa et al., 2018; Correia et al., 2007; Ferreira et al., 2016; Qiang & Cheng, 2019; Yin et al., 2018). Other studies have also found that exposure to MPs may be associated with physiological changes that have the potential to affect individual behavior, such as sex hormone imbalances and decreases in overall reproductive fitness (Wang et al., 2019), changes in the expression of genes related to oxidative stress and inflammation (Assas et al., 2020; Qiang & Cheng, 2019; Rochman et al., 2014), and neurotoxic implications (Mak et al., 2019). Fewer studies have examined the effects of MPs on the outcomes of intraspecific or interspecific interactions (Rios‐Fuster et al., 2021; Sarasamma et al., 2020), but exposure‐induced changes in both predator–prey interactions (Ferreira et al., 2016) and conspecific interactions (Yin et al., 2018) have also been reported.

However, our results are consistent with those of some previous studies that specifically considered intraspecific aggression as a biological marker of MP exposure. For example, Rios‐Fuster et al. (2021) found that gilthead seabream (*Sparus aurata*) exposed to MPs were bolder and showed changes in feeding behavior. However, during competition for food, exposed fish did not differ in the frequency of aggressive acts performed compared with controls. Similarly, Critchell and Hoogenboom (2018) found that exposure to MPs impacted the growth and condition of juvenile *Acanthochromis polyacanthus*, but it had no effect on the frequencies of aggressive behaviors performed by dominant individuals toward subordinates while feeding. By contrast, Sarasamma et al. (2020) found that zebrafish aggression—measured as a percentage of time spent biting at a mirror reflection—decreased following nanoplastic exposure after ~30 days.

What might explain the results of our study? One possibility that cannot be excluded is that, at current environmental levels, the natural ingestion of MPs by freely foraging fish results in body loads below that required for aggressive impairment. Despite being exposed to a concentration of MPs on the high end of reported values (Burns & Boxall, 2018; Cunningham & Sigwart, 2019; Leslie et al., 2017), the fish in our preliminary analyses were found to contain an average of 17–20 MPs in their tissues after 4 or 7 days of dietary exposure. These values are somewhat higher than mean microplastic abundances recovered from fish in three major tributaries of Lake Michigan across a wide range of MP concentrations in surface water samples (10–13 particles per fish at concentrations of 5–90 MP/L; McNeish et al., 2018). Additional support for this explanation comes from the observation that males exposed to a combination of MPs and estrogen did not differ in behavior compared to control males, even though exposure to estrogenic EDCs is well known to reduce levels of circulating androgens in male fish (Salierno & Kane, 2009), with associated reductions in aggression and competitive ability (Bell, 2001; Colman et al., 2009; Saaristo et al., 2009)—including in fathead minnows (Ward et al., 2017).

A second potential explanation for our results could be that the interactions between organisms, MPs and environmental contaminants are more complex than initially assumed. Interestingly, although numerous studies have shown that environmental contaminants sorbed to MPs leach into the tissues of fish following ingestion (Granby et al., 2018; Lee et al., 2019; Rainieri et al., 2018), MPs have also been reported to transfer chemical pollutants out of organisms in a similar manner (Scopetani et al., 2018). The double role played by MPs as a transport mechanism for EDCs could potentially mitigate the impacts of the chemicals sorbed to MP surfaces.

Alternatively, MPs and EDCs may have antagonistic effects in the body or affect different parts of the same system, thereby reducing the organismal consequences of each individual chemical (Hermens et al., 1985; Jackson et al., 2015; Kortenkamp, 2014; Scott & Sloman, 2004). Dang and Wang (2011) showed that mixed modes of action in various selenium and mercury contaminants could cause chemicals to act against or modify the effects of one another in juvenile jarbua terapon (*Terapon jurbua*). Similarly, when exposed to the common pesticides chlorpyrifos and dichlorvos, or a mixture of the two chemicals, mrigal (*Cirrhinus mrigala*) juveniles showed behavioral changes with single chemical exposures, but the mixture had antagonistic effects (Kunwar et al., 2021).

A third potential explanation for our results is that the levels of circulating androgens in our focal male fish were sufficient to offset the estrogenic effects of exposure to MPs and EE2. In this study, we used only highly dominant, reproductively motivated males in trials to minimize the confounding effect of male status on the outcomes of territorial interactions. However, in fish, levels of circulating androgens associated with aggression and dominance (Taves et al., 2009) are highest during spawning and in dominant (e.g., territory‐holding) individuals (Borg, 1994; Parikh et al., 2006). Indeed, a recent meta‐analysis showed that the responses of fathead minnow males to some chemical exposures may differ with respect to social status (Ianova et al., 2017). The authors suggested that subordinate fish could be more sensitive to contaminants than dominant fish due to differences in the interaction between environmental stressors and the endocrine axes that mediate reproduction and responses to stress. Additional research is needed to determine if the independent and synergistic effects of MPs and estrogenic EDCs on aggressive interactions might vary across social dominance hierarchies.

Last, we note that the sample sizes in this study were limited, which may have obscured subtle effects of MP exposure on behavior that would become evident with a larger study. Nonetheless, the results of this study add to the existing body of knowledge about the biological effects of MPs on aquatic biota in three ways; first, despite growing evidence about the adverse effects of exposure on individuals, we know little about the effects of exposure on the outcomes of intraspecific social interactions. Second, by explicitly testing the vector hypothesis, our results shed new insight into the independent and synergistic effects of multiple environmental stressors on individuals. Third, to date, most of our knowledge on microplastic pollution has been conducted on marine systems; it is widely recognized that similar data for freshwater systems is lacking (McNeish et al., 2018; Wagner et al., 2014). We suggest that more studies are needed to robustly predict the short‐ and long‐term ecological and evolutionary effects of anthropogenic environmental change in vulnerable aquatic ecosystems.

## CONFLICT OF INTEREST

None declared.

## AUTHOR CONTRIBUTIONS

**Ally Swank:** Conceptualization (equal); Data curation (equal); Funding acquisition (supporting); Methodology (equal); Visualization (equal); Writing – original draft (equal); Writing – review & editing (equal). **Kadijah Blevins:** Data curation (equal); Investigation (equal); Visualization (equal); Writing – review & editing (equal). **Abby Bourne:** Data curation (equal); Investigation (equal); Writing – review & editing (equal). **Jessica Ward:** Conceptualization (equal); Data curation (equal); Formal analysis (lead); Funding acquisition (lead); Methodology (equal); Project administration (lead); Supervision (lead); Writing – original draft (lead); Writing – review & editing (equal).

## Data Availability

Raw data are available from *Dryad* (https://doi.org/10.5061/dryad.6q573n60z) or from the corresponding author upon request.
